# Electronic Nicotine Delivery System flavor use over time by age group in the US: A longitudinal analysis

**DOI:** 10.18332/tid/162365

**Published:** 2023-05-19

**Authors:** Bekir Kaplan, Jeffrey J. Hardesty, Kevin Welding, Alison B. Breland, Thomas Eissenberg, Joanna E. Cohen

**Affiliations:** 1 Institute for Global Tobacco Control, Johns Hopkins Bloomberg School of Public Health, Baltimore, United States; 2 Center for the Study of Tobacco Products, Virginia Commonwealth University, Richmond, United States

**Keywords:** e-cigarette, flavor, trend

## Abstract

**INTRODUCTION:**

The prevalence of flavor use in Electronic Nicotine Delivery Systems (ENDS) has been assessed in numerous studies, but limited research has focused on flavor use trends and maintenance of flavor preference over time. This study investigated the general trends and maintenance of ENDS flavor use for youth (aged 12–17 years), young adults (aged 18–24 years), and older adults (aged ≥25 years) between 2014 and 2019.

**METHODS:**

Population Assessment of Tobacco and Health (PATH) Study Wave 2 (2014–2015), Wave 3 (2015–2016), Wave 4 (2016–2017), and Wave 5 (2018–2019) youth and adult data were used. Cross-sectional flavor use prevalence (trends) and flavor maintenance (using the same flavor category in at least three consecutive waves) were assessed.

**RESULTS:**

The most reported primary flavor category was fruit among all age groups in all waves. Candy/desserts in waves two, three, four, and menthol/mint in wave five were the second most reported flavor in all age groups. The highest increase was observed for menthol/mint use among youth between wave two (21.9%) and five (58.1%) (OR=5.33; 95% CI: 3.58–7.96). Overall, 37.6% of fruit flavor users, 25.3% of candy/desserts users, 32.0% of menthol/mint users, and 33.4% of tobacco flavor users, maintained use of the same flavor in at least three consecutive waves.

**CONCLUSIONS:**

Fruit flavor had the highest percentages of use and maintenance between 2014 and 2019. While the maintenance of fruit and candy/desserts flavors were higher among youth, adults had substantially higher maintenance percentages for menthol/mint and tobacco flavor. There was a substantial increase in menthol/mint use in wave five among youth, which may affect ENDS flavor maintenance patterns in the future. Understanding maintenance of flavors over time can inform regulation of ENDS flavors.

## INTRODUCTION

Electronic Nicotine Delivery Systems (ENDS) were first marketed in 2007 in the US and have become prevalent, particularly among youth^1,2^. ENDS use prevalence in the US increased from 1.5% in 2011^1^ to 23.6% in 2020^2^ among high school students (past 30-day), and from 1.9% in 2012^3^ to 4.5% in 2019^4^ among adults. In 2017, more than fifteen thousand ENDS flavors were available online^5,6^. Flavors may contribute to increased appeal and palatability, making ENDS easier for new users to initiate^7,8^.

Flavors are one of the most appealing features of ENDS^9-11^ and a leading reason to use ENDS^12-14^. In addition, initiating tobacco use with a flavored product is associated with continued tobacco product use^15,16^ and a substantial proportion of youth and young adult ENDS users stated that their ENDS were flavored when they initiated ENDS use^14^. Among high school students who are current ENDS users, non-tobacco flavored ENDS use increased from 72.2% in 2019^17^ to 84.7% in 2021^18^. Given the substantial use of flavored ENDS among US youth, the U.S. Food and Drug Administration (FDA) issued a ban on unauthorized flavored cartridge-based ENDS products in February 2020, excluding tobacco and menthol flavors^19^.

To date, numerous studies have assessed current flavor use among ENDS users and reported that the most popular ENDS flavors were fruit and candy among youth^16-18,20,21^. Among adults, fruit, candy, and tobacco flavors were the mostly preferred ENDS flavor^21,22^. Although there is much literature reporting ENDS flavor use, limited research has focused on the trends or maintenance of flavor preference over time by age group. Du et al.^23^ assessed 383 adult ENDS users and reported a decrease in tobacco and mint flavor use and a migration toward sweet flavor between 2012 and 2019. In another study^24^ funded by a foundation that is sponsored by a tobacco company, tobacco flavor ENDS use decreased among US adults from 2011 to 2016. To extend knowledge in this area, the overall goal of this study was to assess the trends and maintenance in flavor preference between 2014 and 2019 among US youth, young adults, and adults. Assessing maintenance in flavor preference will also provide insights about the percentages of ENDS users who changed their ENDS flavor over time. Assessing the trends in ENDS flavors and maintenance, as an indicator of changes in flavor preferences, allows us to better understand flavor use in the US and may provide insight into potential benefits and unintended consequences of future flavor regulations.

## METHODS

### Data source

This study uses publicly available youth and adult data from Wave 2 (2014–2015), Wave 3 (2015–2016), Wave 4 (2016–2017), and Wave 5 (2018–2019) of the PATH Study^25^, a large, US nationally representative, cohort study designed to collect information about use of various tobacco products including cigarettes and ENDS, tobacco dependence, cessation, perceptions of risk and harm, overall physical and mental health, peer and family influences, other substance use, and demographic information^25,26^. Information on the sampling procedures can be found in the PATH User Guide^25^.

### Study participants

This study included current adults and youth ENDS users. Current adult ENDS users were defined as respondents who currently use ENDS every day or some days. ‘Current use’ of ENDS among youth was defined as using ENDS within the past 30 days^25^.

### Measurement of ENDS flavor use

Flavor use in ENDS was assessed with the following question: ‘What flavor is/was your regular brand/the brand you last used? (Choose all that apply)’. The response options in wave two consisted of menthol or mint; clove or spice; fruit; chocolate; an alcoholic drink (such as wine, cognac, margarita or other cocktails); candy, desserts or other sweets (hereafter: candy/desserts); or some other flavors. The flavor options ‘tobacco’ and ‘non-alcoholic drink’ (such as coffee, soda, energy drinks, or other beverages) were added to the survey starting in wave three. Participants could select more than one flavor (concurrent use).

### Statistical analysis

*Cross-sectional analyses*

A cross-sectional analysis was first conducted to assess the prevalence of ENDS flavor use by age and wave. Participants who stated at least one ENDS flavor in a wave were included in the cross-sectional analysis. Among all ENDS only users, 27% (n=129) in wave two, 1.2% (n=16) in wave three, 1.0% (n=13) in wave four, and 1.6% (n=31) in wave five, did not choose any ENDS flavor and were excluded from the sample.

*Longitudinal analyses*

Mixed effect logistic regression

Relationships between flavor use across age groups and waves were modeled with mixed effects logistic regression models in longitudinal design using PATH Study waves two, three, four, and five, using a random intercept by survey participant to account for repeated measures over waves. The following covariates were included in the longitudinal regression analysis: age (12–17; 18–24; ≥25 years); sex (male; female); and race/ethnicity (Non-Hispanic White; Non-Hispanic Black; Hispanic; other).

Assessment of maintenance

In addition to longitudinal regression analysis, a subsample of the participants who stated an ENDS flavor use in at least three consecutive waves was created to measure ENDS flavor maintenance in a longitudinal design. ‘Maintenance’ was defined as using the same flavor in at least three consecutive waves of the PATH Study (waves 2–4, 3–5, or 2–5). Assessing maintenance will also provide insights into the percentages of ENDS users that changed their last used flavor. For this longitudinal analyses to measure maintenance, age groups were classified as: 1) Youth (aged 12–17 years in 3 consecutive waves), 2) Age up youth (a youth who became a young adult at some point during the 3 consecutive waves in which they reported ENDS flavor), 3) Young adults (aged 18–24 years in 3 consecutive waves), 4) Age up young adults (a young adult who became an adult at some point during the 3 consecutive waves in which they reported ENDS flavor); and 5) Adults (aged ≥25 years in 3 consecutive waves).

All analyses were performed using STATA version 15.1 (Statacorp., College Station, TX). PATH Study population weights were used to adjust for the complex study design including oversampling and non-response. Observations were weighted according to the ‘all wave’ survey weights for wave five, which are longitudinal weights. Weighted frequency distributions and chi-squared tests were used to compare the maintenance of flavor use between age groups. To account for the survey design, variance estimation was done using 100 balanced repeated replicates with Fay’s method with epsilon=0.3, as recommended in the PATH user guide^25^. Unweighted counts and weighted percentages are presented in the tables. Further information on the weighting procedure can be obtained from the PATH Study Public-Use Files. All tests were two-sided with significance level set at 5%. The current study is a secondary data analysis of existing data; therefore, it was exempt from review by the Johns Hopkins Bloomberg School of Public Health Institutional Review Board.

## RESULTS

### Cross-sectional results

A total of 1380 wave two, 3737 wave three, 3551 wave four, and 5743 wave five participants reported at least one flavor use in the corresponding waves (Table 1). Adults (aged ≥25 years) accounted for over 60% of the sample, while youth accounted for <10% in all waves. The percentages of male participants were slightly over 50% in all waves. Overall, the majority of participants were Non-Hispanic White. More than half of the participants were ‘Some days’ ENDS users and current cigarette users (Table 1).

**Table 1:** Sociodemographic characteristics of the PATH Study Wave 2, 3, 4, and 5 youth and adult cross-sectional participants*, US, 2014–2019

*Characteristics*	*Wave 2 (N=1380) (2014–2015)*		*Wave 3 (N=3737) (2015–2016)*		*Wave 4 (N=3551) (2016–2017)*		*Wave 5 (N=5743) (2018–2019)*	
	*n*	*% (SE)*	*n*	*% (SE)*	*n*	*% (SE)*	*n*	*% (SE)*
**Sex**								
Male	677	53.7 (1.5)	1913	54.6 (0.9)	1847	55.8 (1.0)	2995	54.6 (0.8)
Female	702	46.3 (1.5)	1823	45.4 (0.9)	1704	44.2 (1.0)	2740	45.4 (0.8)
**Age** (years)								
12–17	228	9.3 (0.6)	307	4.3 (0.3)	347	5.4 (0.3)	734	7.5 (0.3)
18–24	396	24.0 (1.2)	1360	28.0 (0.8)	1280	26.9 (0.8)	2515	31.9 (0.7)
≥25	756	66.6 (1.4)	2070	67.7 (0.8)	1924	67.8 (0.9)	2494	60.6 (0.7)
**Race/ethnicity**								
Non-Hispanic White	941	73.2 (1.4)	2299	67.7 (1.0)	2209	68.3 (1.1)	3406	66.0 (0.8)
Non-Hispanic Black	103	7.4 (0.8)	402	9.9 (0.6)	362	9.8 (0.6)	593	10.5 (0.6)
Hispanic	210	11.4 (0.8)	703	14.7 (0.7)	676	15.0 (0.8)	1157	15.9 (0.7)
Other	126	8.0 (0.9)	333	7.7 (0.6)	304	6.9 (0.5)	482	7.5 (0.4)
**ENDS use**								
Every day	409	42.7 (1.8)	648	32.1 (1.3)	672	37.1 (1.4)	1331	38.3 (1.0)
Some days	686	57.3 (1.8)	1616	67.9 (1.3)	1514	62.9 (1.4)	2601	61.7 (1.0)
**Cigarette use status**								
Current	790	66.7 (1.5)	1961	70.7 (1.1)	1753	67.6 (1.1)	2005	56.8 (1.0)
Former	297	30.2 (1.5)	633	26.0 (1.0)	633	28.4 (1.1)	1020	32.2 (0.9)
Never	62	3.1 (0.4)	149	3.3 (0.4)	192	3.9 (0.3)	724	11.1 (0.4)

* Participants stated at least one ENDS flavor use in each wave.

**Figure 1:**
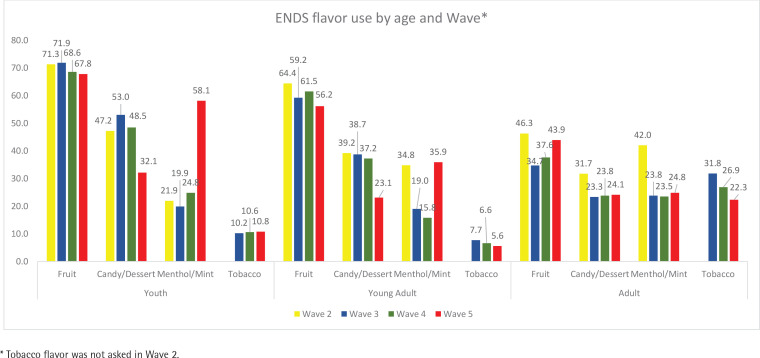
Cross-sectional percentages of current ENDS flavor use by age and wave in PATH Study, 2014–2019

Figure 1 presents the participants’ current primary ENDS flavor use cross-sectionally. The most reported flavor category for the primary flavor used was fruit among all age groups in all waves. The percentages of fruit flavor use across waves ranged 67.8–71.9% among youth, 56.2–64.4% among young adults, and 34.7–46.3% among adults (Figure 1).

Among youth, the second most reported flavor was candy/desserts in waves two, three, and four, ranging 47.2–53.0%. However, in wave five, menthol/mint flavor was the second most reported flavor, reported by 58.1% among youth (Figure 1).

Among young adults, as with youth, candy/desserts flavor was the second most reported flavor in waves two, three, and four (37.2–39.2%) but in wave five, menthol/mint became the second most reported flavor (35.9%) (Figure 1).

Among adults, candy/desserts, menthol/mint, and tobacco flavors in waves three, four, and five had similar percentages and ranged 22.3–31.8% (Figure 1).

The percentages of current use of all other flavors, among all age groups, were <15% in all waves. In addition, among fruit flavor users, candy/dessert flavors were the most concurrent used flavor in all waves with 38.7% in wave two, 32.0% in wave three, 28.1% in wave four, and 21.6% in wave five. ENDS flavor use percentages were similar for ENDS only and dual users.

### Longitudinal results

*Mixed effect logistic regression*

Fruit flavor use was significantly higher among youth (OR=3.37; 95% CI: 2.94–3.86) and young adults (OR=2.16; 95% CI: 1.96–2.38) compared to adults, after adjustment for sex, race/ethnicity, and wave in the mixed effect logistic regression model (Supplementary file Table 1).

Compared to wave two, menthol/mint use among youth significantly increased in wave five (OR=5.33; 95% CI: 3.57–7.96), after adjustment for sex and race/ethnicity in the mixed effect logistic regression model (Supplementary file Table 2).

The percentages of tobacco flavor use among adults in waves three, four, and five were above 20%, and tobacco flavor use was significantly higher among adults compared to youth (OR=3.00; 95% CI: 2.50–3.60) and young adults (OR=5.18; 95% CI: 4.33–6.20), after adjustment for sex, race/ethnicity, and wave in the mixed effect logistic regression model (Supplementary file Table 1). In contrast, candy/dessert flavor use was significantly higher among youth (OR=2.29; 95% CI: 1.95–2.69) and young adults (OR=1.52; 95% CI: 1.36–1.69) compared to adults, in the same model (Supplementary file Table 1).

*Assessment of maintenance over time*

In the subsample of the participants for the longitudinal analysis, 744 wave two, 1636 wave three and four, and 1501 wave five participants reported ENDS flavor use in at least three consecutive waves. Among these participants, in three consecutive waves, 68 (2.1%) remained as youth, 66 (2.3%) became age up youth, 507 (23.5%) were young adults, 67 (3.6%) became age up young adults, and 931 (68.5%) were adults. The other sociodemographic characteristics of the participants in the subsample were similar to the overall sample.

Overall, among all age groups, 37.6% of fruit flavor users, 25.3% of candy/desserts users, 32.0% of menthol/mint users, and 33.4% of tobacco flavor users, maintained use of the same primary flavor in at least three consecutive waves (Table 2). In other words, more than 60% of fruit flavor users and two in three candy/desserts, menthol/mint, and tobacco flavor users changed their primary ENDS flavor category by switching to other flavors during this period. Youth had the highest fruit flavor maintenance percentage (54.1%) compared to other age groups, but this percentage was not significantly different than other age groups’ fruit flavor maintenance percentages (p=0.285). However, there were substantial differences in the percentage of respondents who maintained use of menthol/mint and tobacco flavors across age groups. While 38.8% of adults maintained tobacco flavor use, only 5.9% (n=1) of youth and none of the age up youth maintained tobacco flavor use. As with tobacco flavor, 44.2% of adults maintained their use of menthol/mint flavor but this percentage was 3.6% (n=2) for youth and 15.3% (n=3) for age up youth (Table 2).

**Table 2:** Percentages of participants (aged ≥12 years) who maintained use of the same primary flavor in at least three consecutive waves of the PATH Study Waves 2, 3, 4, and 5, US, 2014–2019

*Age groups*	*Fruit*		*Candy*		*Menthol/mint*		*Tobacco*	
	n (N)*	*%*	n (N)*	*%*	n (N)*	*%*	n (N)*	*%*
**Youth** (All waves)	36 (68)	54.1	14 (55)	25.0	2 (47)	3.6	1 (15)	5.9
**Age up youth**	23 (61)	35.7	9 (52)	18.0	3 (33)	15.3	0 (13)	0.0
**Young adults** (All waves)	179 (465)	38.8	91 (375)	25.2	30 (235)	13.6	6 (70)	7.5
**Age up young adults**	20 (58)	36.3	4 (42)	10.2	8 (37)	13.9	1 (14)	2.6
**Adults** (All waves)	226 (615)	36.5	116 (474)	26.7	141 (349)	44.2	121 (332)	38.8
p**	0.285		0.171		<0.001		<0.001	
**Total**	484 (1267)	37.6	234 (998)	25.3	184 (701)	32.0	129 (444)	33.4

* N: number of participants who responded either ‘Yes’ or ‘No’ to the corresponding flavor question in at least 3 consecutive waves. n: number of participants who responded ‘Yes’ to the corresponding flavors in at least 3 consecutive waves.

** Chi-squared test.

## DISCUSSION

This study is one of few longitudinal analyses to assess ENDS flavor use trends and maintenance in ENDS flavor preference. To the best of our knowledge, this is the first study to report a substantial increase in menthol/mint use among youth after 2018 in wave five. Compared to previous PATH Study waves, menthol/mint use almost tripled among youth in wave five (December 2018 – November 2019). This increase is likely because JUUL Labs, maker of one of the most reported ENDS brands used among youth at the time^18^, stopped the retail sale of several non-tobacco and non-menthol flavored pods such as mango, fruit, cucumber, and creme in November 2018, one month before the PATH Study wave five data collection began^27^. As a response, youth JUUL users might have switched to menthol/mint flavored pods as the only available non-tobacco JUUL flavor. The increase in menthol/mint use in wave five and concurrent use of different flavor categories demonstrates that with partial restrictions on ENDS flavors, ENDS users will switch to other non-exempt flavors or to brands for which the restrictions do not apply.

The percentage of maintaining ENDS flavors at least three years remained <40% for all flavor categories, which suggests that most of ENDS users switched their ENDS flavors. Despite the low maintenance percentages, ENDS users were more likely to maintain fruit flavor use over at least three years, and less likely to maintain use of other ENDS flavors over this time period. Moreover, the percent of ENDS users who maintained flavor use varied by age group and ENDS flavor types. For instance, maintaining fruit flavor was higher among youth; however, maintaining menthol/mint or tobacco flavors were higher among adults compared to other age groups. High non-tobacco flavor use and a high proportion of flavored ENDS among first time ENDS users have been reported in the literature^14,18^. This study contributes to the scant literature about maintenance in ENDS flavor preference. Results suggest that, over time, more than half of youth and almost two in five other age groups maintained the use of fruit flavor category between 2014 and 2019. The high maintenance percentage in fruit flavor and low maintenance percentage in tobacco flavor, especially among youth and young adults, is consistent with previous studies that reported a decrease in tobacco flavor use and increase in fruit flavors^23,24^. ENDS flavor has important role in the initiation of ENDS use, especially among youth^12^, with a high percentage of maintaining fruit flavor use. The results of the current nationally representative longitudinal study extend the current literature that flavors could be a contributing factor for continuing to use ENDS.

Maintaining use of tobacco flavor in this study was more than double among adults compared to youth and young adults. Two in five adults maintained use of tobacco flavored ENDS, whereas this proportion was one in seven among youth or young adults. One explanation could be that some adults might use tobacco flavor ENDS because they have a taste that is similar to combustible cigarettes, whereas youth might use other flavors for a more palatable taste of ENDS^28^.

### Strengths and limitations

Assessing ENDS flavor maintenance by age group in a longitudinal design is the strength of this study. This study has, however, several limitations. First, the PATH survey assesses the last used ENDS flavor; however, some ENDS users might change the flavor category in their ENDS many times between waves. In addition, the last used flavor might be different than the most used flavor. Second, some ENDS flavors might have been classified in another flavor category by their users; for example, a fruit flavor might have been specified in candy/dessert category or vice versa. Therefore, the actual flavor might not have been chosen for those participants, which might affect the percentages of flavor use and maintenance. Third, the regulatory action on flavored pods could have significantly reshaped current flavor preferences, which would only be evident in PATH wave 6. Fourth, tobacco flavor was first asked in wave three of the PATH Study, therefore, we were unable to assess the maintenance or change in tobacco flavor and its addition may have affected the proportion of other flavors selected.

## CONCLUSIONS

There was a substantial increase in menthol/mint flavor use in wave five and most of the ENDS users did not maintain (or switched) their ENDS flavors. Maintaining use of fruit flavor in at least three consecutive waves of the PATH Study between 2014 and 2019 was higher compared to the maintenance of use of other flavors, and maintaining use of tobacco flavor was substantially higher among adults compared to other age groups. These data suggest a portion of users may have durable flavor category preferences and others may be more willing to at least try new flavor categories. While further exploration is warranted, a partial or comprehensive flavor ban may differentially impact the likelihood that each group of users will switch flavor categories or quit using ENDS. In addition, further research is needed to assess the effect of the FDA’s recent ENDS flavor ban in February 2020.

## Data Availability

The data supporting this research is available from the following link: https://www.icpsr.umich.edu/web/NAHDAP/studies/36498/datadocumentation
