# Supplementary material for: Electronic Nicotine Delivery System flavor use over time by age group in the US: A longitudinal analysis
Source: Tob Induc Dis. 2023 May 19;21:67. doi: 10.18332/tid/162365 (PMC10198257; doi:10.18332/tid/162365)
Supplement: Supplementary file 1 [file TID-21-67-s1.pdf]

Supplemental Table 1: Odds Ratios of ENDS flavor use by PATH Study waves and age groups					
**Variables**	**Groups**	**Fruit**	**Candy**	**MM**	**Tobacco**
Waves	2	1 (Ref)	1 (Ref)	1 (Ref)	NA
	3	0.67 (0.58-0.77)	0.79 (0.67-0.93)	0.46 (0.39-0.53)	(Ref)
	4	0.75 (0.65-0.86)	0.78 (0.67-0.90)	0.43 (0.38-0.50)	0.80 (0.72-0.89)
	5	0.81 (0.70-0.93)	0.60 (0.52-1.43)	0.69 (0.59-0.80)	0.64 (0.57-0.71)
Age Groups (Years)	≥25	1 (Ref)	1 (Ref)	1 (Ref)	3.00 (2.50-3.60)
	12-17	3.37 (2.94-3.87)	2.29 (1.95-2.69)	1.57 (1.37-1.80)	1 (Ref)
	18-24	2.16 (1.96-2.38)	1.52 (1.36-1.69)	1.01 (0.89-1.15)	0.58 (0.45-0.73)

Supplemental Table 2: Odds Ratios of ENDS Flavor use by PATH Study wave among each age groups				
**Flavors**	**Waves**	**Youth**	**Young Adults**	**Adults**
Fruit	**2**	1 (Ref)	1 (Ref)	1 (Ref)
	**3**	1.03 (0.67-1.58)	0.79 (0.63-0.98)	0.61 (0.51-0.73)
	**4**	0.87 (0.59-1.10)	0.87 (0.70-1.09)	0.69 (0.58-0.82)
	**5**	0.87 (0.61-1.23)	0.69 (0.55-0.87)	0.87 (0.73-1.04)
Candy	**2**	1 (Ref)	1 (Ref)	1 (Ref)
	**3**	1.29 (0.88-1.88)	1.00 (0.78-1.29)	0.67 (0.53-0.83)
	**4**	1.06 (0.74-1.53)	0.94 (0.74-1.20)	0.89 (0.56-0.85)
	**5**	0.52 (0.36-0.75)	0.47 (0.37-0.58)	0.70 (0.58-0.84)
MM	**2**	1 (Ref)	1 (Ref)	1 (Ref)
	**3**	0.91 (0.58-1.41)	0.44 (0.34-0.57)	0.41 (0.34-0.50)
	**4**	1.17 (0.79-1.74)	0.35 (0.26-0.48)	0.40 (0.34-0.48)
	**5**	**5.33 (3.57-7.96)**	1.03 (0.77-1.38)	0.42 (0.35-0.50)
Tobacco	**2**	NA	NA	NA
	**3**	1 (Ref)	1 (Ref)	1 (Ref)
	**4**	1.04 (0.65-1.67)	0.84 (0.60-1.17)	0.79 (0.70-0.89)
	**5**	1.05 (0.63-1.75)	0.70 (0.53-0.92)	0.62 (0.54-0.71)

© **2023 Kaplan B. et al.**
